# Consensus building around nutrition lessons from the 2014–16 Ebola virus disease outbreak in Guinea and Sierra Leone

**DOI:** 10.1093/heapol/czy108

**Published:** 2019-02-07

**Authors:** Stephen R Kodish, Aline Simen-Kapeu, Jean-Max Beauliere, Ismael Ngnie-Teta, Mohammed B Jalloh, Solade Pyne-Bailey, Helen Schwartz, James P Wirth

**Affiliations:** 1 GroundWork, Hintergass 1, Fläsch, Switzerland; 2 UNICEF West and Central Africa Regional Office, Yoff, Dakar, Senegal; 3 UNICEF Guinea Country Office, Conakry, Republic of Guinea; 4 FOCUS 1000, 15 Main Motor Rd, Brookfields, PMB 393, Freetown, Sierra Leone; 5 Ministry of Health and Sanitation, Youyi Bldg, Brookfields, PMB 393, Freetown, Sierra Leone

**Keywords:** Nutrition, participatory research, stakeholders, Ebola virus, qualitative research

## Abstract

There are important lessons learned from the 2014–16 Ebola virus disease outbreak in West Africa. However, there has not been a systematic documentation of nutrition lessons specifically. Therefore, this study sought to generate multiple stakeholder perspectives for understanding the nutrition challenges faced during the Ebola virus disease outbreak, as well as for consensus building around improved response strategies. Participatory workshops with 17 and 19 participants in Guinea and Sierra Leone, respectively, were conducted in February 2017. Workshops followed the Nominal Group Technique, which is a methodological approach for idea generation and consensus building among diverse participants. Those findings were triangulated with qualitative interview data from participants representing government, United Nations bodies, civil society, non-governmental organizations and local communities in both Guinea (*n* = 27) and Sierra Leone (*n* = 42). (1) *Reduced health system access and utilization, Poor caretaking and infant and young child feeding practices*, *Implementation challenges during nutrition response, Household food insecurity* and *Changing breastfeeding practices* were five nutrition challenges identified in both Guinea and Sierra Leone. (2) Between settings, 14 distinct and 11 shared organizational factors emerged as facilitators to this response. In Sierra Leone, participants identified the *Use of Standard Operating Procedures* and *Psychosocial counselling*, whereas in Guinea, *Hygiene assistance* was distinctly important. *Political will, Increased funding, Food assistance* and to a lesser extent, *Enhanced coordination*, were deemed ‘most important’ response factors. (3) The top nutrition lessons learned were diverse, reflecting those of nutrition policy, programme implementation, community activity and household behaviours. Disease outbreaks pose widespread nutrition challenges to populations in resource-constrained settings where global health security is not a guarantee. These findings should be considered for emergency nutrition preparedness and inform evidence-based priority setting in the post-Ebola virus context of Guinea and Sierra Leone.

**Key Messages**

- Guinea and Sierra Leone experienced many similar multi-level impacts on infant and young child nutritional status resulting from the Ebola virus disease (EVD) outbreak in 2014–16.
- Stakeholders who responded to population-level nutrition needs during the EVD outbreak explained key organizational factors that made the response more effective over time.
- Salient ‘nutrition lessons learned’ were described by stakeholders in Guinea and Sierra Leone and can be used as evidence to inform nutrition recovery and future response strategies post EVD.

## Introduction

The 2014–16 outbreak of Ebola virus disease (EVD) was an ‘international public health emergency’, with nearly 29 000 ‘confirmed, probable and suspected cases’ and >11 000 deaths reported in Guinea, Liberia and Sierra Leone (WHO, 2016). While medical treatment of EVD-infected individuals and containment of the outbreak was the priority of early response efforts, stakeholders [e.g. governments, United Nations (UN) agencies and non-governmental organizations (NGOs)] responded to the food and nutrition needs of EVD patients, survivors and vulnerable groups (e.g. infants and young children) who were directly and indirectly impacted by the outbreak. Considering the outbreak scale, efforts required increased capacity, logistics and resources.

Nutrition-related interventions took several different forms in Guinea and Sierra Leone. In 2014, the World Health Organization (WHO), World Food Programme (WFP) and United Nations Children’s Fund (UNICEF) developed multiple nutrition guidelines to respond to the nutritional needs of the aforementioned population groups (UNICEF *et al.*, 2014; WHO *et al.*, 2014). Also, food assistance was provided in the form of staple foods and specialized nutritious foods, such as ready-to-use therapeutic foods for children with acute malnutrition. Social and behaviour change communications (SBCC) complemented these nutrition-specific interventions to reduce stigma, enhance health-seeking behaviours, improve infant and young child feeding (IYCF) practices, and mobilize entire communities in Community-based Management of Acute Malnutrition (CMAM) programmes.

The EVD outbreak had nonetheless far-reaching impacts on an already suboptimal food security situation before the outbreak, where 33–50% of households had already been ‘vulnerable to food insecurity’ in Guinea and Sierra Leone, respectively (WFP, 2015). During the outbreak, food security was impacted, including decreased food availability from lower agricultural yields, market closures, trade restrictions and bans affecting animal-source protein consumption, such as bushmeat (Plan, 2015; Streifel, 2015). Those foods that were available became largely inaccessible due to higher food prices and travel restrictions limiting market access and trading (Ministry of Agriculture 2014; FAO and WFP, 2015; Elston *et al.*, 2017). Despite this evidence, there is a lack of clarity around the priority areas for developing improved nutrition responses in the future, an important consideration during humanitarian responses when resources and capacity are finite.

This study therefore sought to garner stakeholder perspectives and build consensus around both the key nutrition-related challenges faced and the important lessons to inform strategies for addressing nutrition in future outbreaks. We compared findings from Guinea and Sierra Leone to: (1) identify and prioritize the main nutrition-related challenges faced during the EVD outbreak; (2) understand the organizational factors that facilitated an improved nutrition response over the course of the outbreak and (3) build stakeholder consensus around key nutrition lessons learned to inform future response strategies.

## Materials and methods

### Design

This participatory study was designed as the final phase in a multi-phased, qualitative study conducted in Guinea and Sierra Leone. This phase had two primary objectives: (1) to provide a forum for presenting qualitative interview findings to stakeholders for their feedback and corroboration, and (2) to build consensus around priority areas that had been identified through previous qualitative work (Kodish *et al.*, 2018).

### Sampling

Participants were identified and recruited by UNICEF in Guinea and by FOCUS 1000, an NGO, in Sierra Leone. They were purposively sampled as representatives of important stakeholder groups involved in the EVD response, including those from government, civil society, NGOs and the UN (Marshall, 1996). Participants were not compensated for their involvement in this study.

### Data collection and procedures

#### Participatory workshops using nominal group technique

In Guinea and Sierra Leone, participatory workshops were held in February 2017 using the Nominal Group Technique (NGT), which is an approach for generating ideas and building consensus around proposed solutions to questions that require judgmental decision-making (Carney *et al.*, 1996). This approach allows different ideas to be shared and systematically synthesized for consensus building among diverse participants (Harvey and Holmes, 2012). In doing so, a multi-step process was followed in each round (Dunham, 1998). Three rounds involving each of the steps below were conducted and led by two facilitators (SRK and JPW).

**Step 1.** A key question was posed to participants

Three primary questions guided the rounds of data collection: (A) What were the foremost challenges related to IYCN during the EVD outbreak? (B) Within your organizations, what were the facilitating factors that let you effectively respond to the nutritional needs of all people during the EVD outbreak? and (C) What are the key nutrition lessons learned from the EVD outbreak?

**Step 2.** Silent generation of ideas individually

After ensuring that workshop participants understood the question content, they were asked to each generate a list of ideas in response to the question individually on his/her own paper. This brainstorming step lasted ∼10 min.

**Step 3.** Recording ideas as a group in a round-robin style of facilitation

Then, the facilitators solicited responses from the workshop participants in a round-robin style of facilitation where any answer given was written on flip charts at the front of the room. Facilitators worked to ensure that all participants shared at least one idea. At this stage, there were no right or wrong answers and ideas were solicited until the group had no more unique suggestions to offer.

**Step 4.** Group discussion to create a master list of items without repetitions or ambiguity

Facilitators made the exhaustive list viewable to all participants. Then working one by one through each idea on the list, facilitators and participants discussed the merits of the ideas, offered explanations and removed any duplicate responses. This lengthy process resulted in a consolidated list of unique responses.

**Step 5A.** Voting on ideas individually

Using the shortened list, each participant then individually chose five responses that they felt were the highest priority items and ranked those in order of importance on an index card. Of the five responses selected by each participant, response 1 was considered the most important and response 5 the least important. The voting was done anonymously without participant names written on the cards.

**Step 5B.** Pile sorting individually

Each round followed these same steps in both countries through the first two questions. For the final question, the same process of independent brainstorming and discussion produced consolidated lists following Steps 1–4 as indicated. However, for question C, a pile sorting approach instead of voting was used in attempt to better capture all brainstormed ideas from Step 4, as voting constrains individuals to only choose five items when they may have wanted to vote for more of them.

Pile sorting is an ethnographic method whereby each participant is asked to sort items based on how similar or different they perceive them to be (Weller and Romney, 1988). After developing a master list of 15 and 21 items (lessons learned) in Sierra Leone and Guinea, respectively, each participant sorted their cards into three piles based on their perception of the relative importance of each nutrition lesson learned from the outbreak: (1) most important, (2) somewhat important and (3) less important. The number corresponding to each item on the back of each card was recorded for analysis.

### Data analysis

#### Tallying votes

After each round of voting, index cards were provided to the workshop facilitators, who aggregated the data. To weight the responses by priority, responses 1, 2, 3, 4 and 5 were given 5, 4, 3, 2 and 1 points, respectively. Items were entered into Microsoft Excel and tallies for all items were made. Prior to presentation of the data, the response options were aggregated using a standard nomenclature to compare the responses and give priority to each response for Guinea and Sierra Leone.

#### Multi-dimensional scaling of pile sorts

The numbers associated with the ‘lessons learned’ items were entered into Anthropac software (Borgatti, 1990). Doing so with Sierra Leone data yielded 15 × 15 item-by-item matrices and in Guinea 21 × 21 matrices, with each cell representing the proportion of times two items were in the same pile. Multi-dimensional scaling (MDS) was then used to analyse these aggregate proximity matrices and the ‘model stress’, a goodness of fit indicator, was calculated for each workshop’s data set (Weller and Romney, 1988). Stress, which ranges from 0 (perfect fit) to 1 (no fit), is the aggregate of the representation errors of each data-distance pair (Borg *et al.*, 2017); in other words, it explains how non perfectly proportional the graph is (Bernard *et al.*, 2016). There is not a concrete threshold for model stress, as many factors influence it, including dimensionality of the MDS plot (a 2D graph would expect higher stress than 3D space), number of items being plotted (the greater the number of points, the higher the stress) and reliability of the data (Borg *et al.*, 2017).

The model stress from Sierra Leone data was 0.14, indicating a ‘reasonable stress’ in two dimensions (Sturrock and Rocha, 2000). For Guinea, data from two participants who did not fit the cultural consensus model were dropped as outliers. Prior to their removal, model stress was 0.24; and following removal, it became 0.19, indicating a better fit without their inclusion. Visual MDS maps were then generated to display results.

#### Qualitative interview data

Findings from the NGT workshops were triangulated with semi-structured, qualitative interview data previously collected by the research team among organizational stakeholders and community members in both Guinea (*n* = 27) and Sierra Leone (*n* = 42). Those methods have been explained in detail elsewhere (Kodish *et al.*, 2018).

### Ethical approval

The office of *Sierra Leone Ethics and Scientific Review Committee* and the Guinea Ministry of Health both approved the study procedures. Participants provided free and informed consent prior to interviews and participation in the workshop was voluntary.

## Results

### Characteristics of participants of NGT workshops

The Guinea and Sierra Leone workshops comprised 17 and 19 participants, respectively. In both workshops, participants represented government agencies, UN agencies and NGOs. In Sierra Leone, four participants were EVD survivors; in Guinea, no EVD survivors participated in the workshop.

### Participant voting and responses to question A

**Question A.** What were the challenges related to IYCN during the EVD outbreak?

Table 1 presents thematic areas representing the primary IYCN challenges as reported by the participants in the NGT workshops in Guinea and Sierra Leone. Between settings, 12 different challenges were identified while five themes were shared: *Health system access and utilization*, *Poor caretaking and IYCF practices*, *Implementation challenges during nutrition response*, *Household food insecurity* and *Changing breastfeeding practices*. Voting to prioritize these challenges resulted in differential findings between country settings.

**Table 1.** Important challenges related to infant and young child nutrition during the EVD outbreak ranked by number of votes cast per item

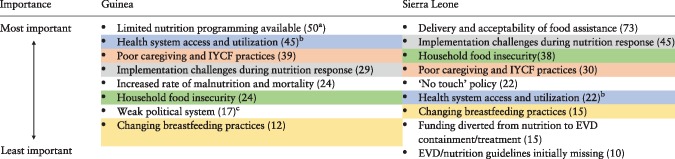		

For colour, please refer online.

a Numbers in parentheses indicate the number of votes cast for each item.

b Same colours indicate similar content areas mentioned between Guinea and Sierra Leone workshops.

c Refers to limited political willpower to improve nutrition, according to workshop participants.

In Guinea only, workshop participants identified the *Weak political system* as a key challenge impacting IYCN during the outbreak. Interview data suggest that the political importance of nutrition increased in response to the outbreak.

*This advocacy* [underscoring the political importance of nutrition] *is understood today and nutrition, I think, has proven its worth in the politics of health in the country and has become a central preoccupation and a major source of interest for many players, particularly the government* (Interview with UN Director, Conakry, Guinea).

A weak political system was not mentioned as a challenge by participants in Sierra Leone. Conversely, respondents identified the ‘*No touch*’ *policy* as a key factor that weakened IYCN in Sierra Leone but not in Guinea. Interview data also reflect this challenge in Sierra Leone:

*The EVD outbreak impacted IYCF because their feeding habits changed during the scourge, as we were warned strongly that if we feel sick, we should not touch our child… not to feed them, but rather go to the hospital for treatment immediately…and also if one is an EVD survivor then we should not breastfeed our babies at all* (Interview with EVD survivor, Western Area, Sierra Leone).

**Question B.** What were the facilitating factors that enabled organizations to effectively respond to the nutritional needs (of all individuals) during the EVD outbreak?

Table 2 presents the factors reported by the participants that enabled organizations to respond to population-level nutritional needs. In total, 14 distinct factors were expressed by participants between workshops, including 11 shared themes. In Sierra Leone, participants uniquely identified the *Use of Standard Operating Procedures (SOPs)* and *Psychosocial counselling*, whereas in Guinea, *Hygiene assistance* was distinctly important:

*… this matter of hand-washing, which was not part of the Guinean service providers’ culture; when caring for each patient you must wash your hands; you must let them get dry and wear gloves before checking another patient…that* [practice] *did not exist. Therefore, when it comes to the assets* [of the organization] *I can firstly note this* [new] *hand-washing culture* (Interview with Hospital Management, Conakry, Guinea).

**Table 2.** Factors that enabled organizations to respond to nutritional needs in Guinea and Sierra Leone

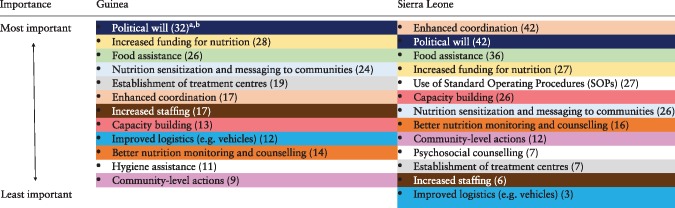

For colour, please refer online.

a Numbers in parentheses indicate the number of votes cast for each item.

b Same colours indicate similar content areas mentioned between Guinea and Sierra Leone workshops.

Participants in both workshops identified *Political will, Increased funding, Food assistance*, and to a lesser extent, *Enhanced coordination*, as among the ‘Most Important’ factors. However, participants in Sierra Leone ranked *Enhanced coordination* as the most important factor, both at the organizational level among specific international bodies and throughout local community networks:

*We were also coordinating with paramount chiefs, section chiefs and other local authorities to ensure that the drafted by-laws were fully implemented. The by-laws covered nutritional support … each time we went to a community to supply food, we were given chiefdom support. And that was a success, because each time we went to the interior villages that were not to be reached by motor vehicle, the chiefdom people would help us carry the* [food] *supply to the people* (Interview with Social Protection Manager, Bo district, Sierra Leone).

Participants in Guinea considered *Political will* to be more important than coordination*.* Also, participants in Sierra Leone identified the *Use of SOPs* as an important factor, whereas this item was not mentioned in Guinea.

**Question C.** What were the key nutrition-related lessons learned from the EVD outbreak?

#### Sierra Leone

Participants categorized ‘nutrition lessons learned’ items (*n* = 15) into three categories based on their perceived importance of those items during response (Table 3).

**Table 3.** Description of clusters based on multi-dimensional analysis of ‘nutrition lessons learned’ items (*n* = 15) in Sierra Leone

Sierra Leone ‘nutrition lessons learned’ items by grouping
**Most important**
Global nutrition support
Adequate funding available for nutrition
Early and appropriate communications
Food provision restricted people’s movements
Nutrition counselling at health facilities
Surveillance system in place to identify malnutrition cases
Establishment of food security network
Political will and policy
Nutritional support to households
Community-level involvement
**Moderately important**
Research-based programme decisions
Strong partnerships
High nutrition capacity and expertise
**Less important**
Mothers’ ability to screen using MUAC
Understanding nutrition needs of survivors

MUAC, mid-upper arm circumference.

The **MOST IMPORTANT** lessons learned include a wide range of nutrition actions at multiple socio-ecological levels, including *nutritional support to households, nutrition counselling at health facilities, community-level involvement* and *political will and policy.* The **MOST IMPORTANT** items traversed levels of influence, with both political/government- and community-level factors sorted into this larger category of lessons learned. Interview data also underscored how multiple aspects of a response are important for addressing nutrition.

*Nutrition is about educating the people so until we need to have personnel to provide the necessary education; we need to provide the necessary communication work with other stakeholders in nutrition. Is not just about health; the food has to* [also] *be there…it’s a multi-sectorial approach … it is about plenty of hands coming together to ensure that you have the nutrition…a multi-stakeholder* [approach] *is what we need if you are talking about nutrition* (Interview with District Government Representative, Bo district, Sierra Leone).

**Figure 1.**
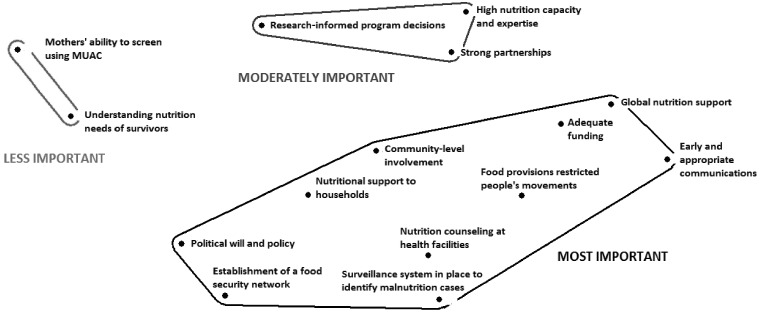
Multi-dimensional scaling map of ‘nutrition lessons learned’ in Sierra Leone (*n* = 15 items)

The **MODERATELY IMPORTANT** lessons included *research-based programme decisions, strong partnerships* and *high nutrition capacity and expertise.***LESS IMPORTANT** items were those only at the individual level and included *mothers’ ability to screen using MUAC* and *understanding nutrition needs of survivors*. The MDS plot resulted in a 2D figure with three distinct clusters (Figure 1).

There was no subcultural variation among participants, i.e. respondents share a single cultural model based on the statistical assessment of the level of agreement within this knowledge domain (Romney *et al.*, 1986) with respect to this domain of ‘nutrition lessons learned’ (eigenvalue: 2.675; eigenratio: 3.469), and the plot had reasonable stress (0.135). The **MOST IMPORTANT** category contained the largest number of items; those included in this group were diverse, reflecting lessons related to governmental policy, programme implementation, community-level activities and household-level responses.

#### Guinea

In Guinea, workshop participants performed a constrained pile sort of ‘nutrition lessons learned’ items (*n* = 21), resulting in three distinct piles representing level of importance given to each nutrition lesson (Table 4).

**Table 4.** Description of clusters based on multi-dimensional analysis of ‘nutrition lessons learned’ items (*n* = 21) in Guinea

Guinea ‘nutrition lessons learned’ items by grouping
**Most important**
Establishment of emergency response contingency plan
Improved coordination of nutrition interventions
Dissemination of emergency nutrition guiding documents
Well-trained health and nutrition capacity
Multi-sectoral nutrition policy
Establishment of disease surveillance system
Strengthened health security
Multi-level communications
Importance of handwashing promotion
**Moderately important**
Awareness of nutrition guidelines
Fear can limit health-seeking behaviours
Nutritional surveillance
Civil society engagement
Nutrition advocacy
Well-equipped health facilities
Nutritional support to households
Community (health workers and members) lacked nutrition awareness
Nutritional management (at Ebola treatment centres) reduced mortality
**Less important**
Organizational support at community level
Nutrition prioritization
Decentralized communications

The **MOST IMPORTANT** lessons learned formed a cluster of nine items that were related to health and nutrition assurance at policy and institutional levels, including but not limited to *multi-sectoral nutrition policy*, *establishment of an emergency response contingency plan* and *dissemination of emergency nutrition guiding documents*. Interview data highlight how each factor is not distinct, however: *improved coordination* includes the mobilization of *well-trained health and nutrition capacity* not only in a centralized, but also in a decentralized health system:

*… there are needs at the central level for coordination … the central level is strategic, as it helps in the* [response] *design, helps the overall* [response] *direction, and also helps in the correction of field activities … Secondly, we trained a lot of human resources. And we realized that we did not have a human resource-related problem during the epidemic because all human resources agreed to go and serve … but come back to the traditional system of the Ministry of Health? It was not possible … therefore, that is one of the lessons we must draw. There must be a system that motivates people and brings them to leave urban centers to rural centers. And for that, partners must play a role accordingly* (Interview with Ministry of Health Representative, Conakry, Guinea).

**Figure 2.**
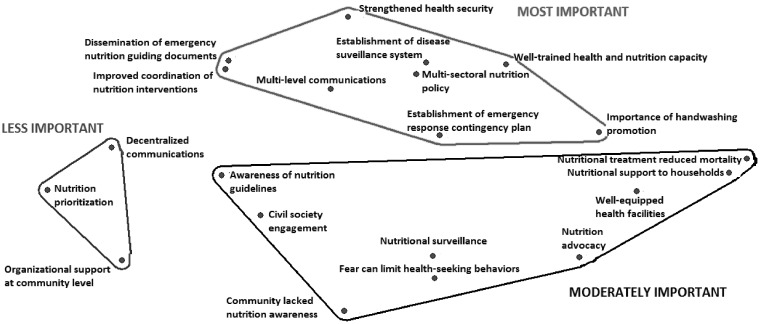
Multi-dimensional scaling map of ‘nutrition lessons learned’ items in Guinea (*n* = 21 items)

The **MODERATELY IMPORTANT** items formed another grouping of nine items related to assurance, but at the community and household levels. For instance, *nutritional support to households* and *well-equipped health facilities* fell into this category. The **LESS IMPORTANT** items consisted of three items, including *organizational support at community levels*, *nutrition prioritization* (during the response) and *decentralized communications*. The 21 items were visually depicted to illustrate the three distinct categorizations (Figure 2).

The MDS plot derived from sorting in Guinea also indicated a strong fit to the cultural consensus model, (eigenvalue: 3.447; eigenratio: 6.216), with moderate stress (0.199).

## Discussion

This study highlights both the multi-level, nutrition-related challenges faced and the important opportunities identified during the EVD outbreak in Guinea and Sierra Leone from the perspectives of diverse, yet interconnected stakeholders who had experienced the event firsthand. Findings underscore the fragility of health and nutrition in these two settings where global health security is not a guaranteed human right to the majority of those populations affected.

Overall, our findings illustrate a range of reasons why food and nutrition security was impacted during the EVD outbreak. They reinforce how the vulnerability of a weak health system and the resultant social and economic impacts that contributed to reduced access to health care and health service utilization may have impacted nutrition-related morbidity and mortality through immediate and underlying factors (Elston *et al.*, 2017). They also underscore key characteristics of effective responses, which include well-planned coordination efforts, integrated approaches encompassing both clinical and community components, and appropriate social and behavioural considerations, to name a few. Now, post-EVD, the Ministries of Health in both Guinea and Sierra Leone, with support from international agencies, are focused on the recovery phase using longer-term, integrated strategies to ensure better food and nutrition security.

### Post-EVD nutrition programming in Guinea and Sierra Leone

First, there is a prioritization on building resilience by reinforcing a multi-sectoral, multi-level and multi-stakeholder systematic approach on nutrition coordination, advocacy and implementation at both the national and decentralized levels. Nutrition implementation challenges, including uninterrupted delivery of food assistance, stemming in part from weak coordination was a primary barrier to a more effective nutrition response found in both this work and others (Elston *et al.*, 2015). In practice, this means that support is moving away from a cluster-based approach, which was implemented to strengthen system-wide preparedness and rapid response interventions during the EVD outbreak. After all, delivery strategies for nutrition interventions in humanitarian emergencies such as EVD necessitate better coordination of key stakeholders, higher prioritization of nutrition actions and stronger integration of interventions across sectors than might be needed in stable circumstances (WFP, 2016; Kiess *et al.*, 2017).

Second, it includes ensuring comprehensive nutrition responses across entire food systems. Efforts are being made to increase local food production to rebuild the livelihoods of affected households and to scale up nutrition-sensitive programmes for building more resilient communities–strategies that are aligned with and supported by the Scaling Up Nutrition movement (WHO, 2017). In the early EVD response, preparations were made to address a high burden of acute malnutrition focused on facility-based therapeutic care, targeted/blanket supplementary feeding and the provision of micronutrient supplements. Indeed, we found food assistance to be an important aspect of this response. To be sure, these nutrition-specific interventions should have served as entry points that supported, rather than supplanted nutrition-sensitive actions seeking to address underlying causes of poor nutrition. In fact, caseloads of acute malnutrition did not rise as expected during the outbreak so community-based approaches became an increasingly important part of the response over time. Effective interventions employed in response to the AIDS epidemic, although different from EBV, have yielded positive outcomes using both food assistance and livelihood support in concert (Aberman *et al.*, 2014).

Third, a renewed focus is on embracing SBCC as an effective approach for raising health and nutrition awareness, enhance nutrition knowledge and attitudes and increasing demand for nutrition services using tailored and intensified messaging in combination with community mobilization. SBCC for nutrition has become a bigger priority globally, using both interpersonal approaches and mass media for effective nutrition responses, even in emergencies (Gillespie *et al.*, 2016). The social and behavioural dimensions of this outbreak have been well documented (Streifel, 2015; Elston *et al.*, 2017; Jalloh *et al.*, 2017). Not surprisingly then, this study and others found that the SBCC efforts—despite being beset by early shortfalls—eventually played a critical role to improve health- and nutrition-seeking behaviours necessary for stemming the outbreak (Bedrosian *et al.*, 2016; Jalloh *et al.*, 2017). More national-level resources are therefore being put into community-nutrition support networks for caregivers, as well as increased nutrition counselling services in health facilities. Lessons learned from this situation and others suggest that communities benefit from empowerment to become the main drivers of health promotion and nutrition-related behaviour change, even in emergency situations among crisis affected populations (Hall *et al.*, 2011).

Fourth, strengthening institutional capacities, focused on using community-based systems, has become more important post EVD. Community-based nutrition platforms offer a unique opportunity to better engage and reach vulnerable populations living in poverty (Doherty *et al.*, 2010). As community health worker programmes are increasingly being adopted as effective ways to address maternal, newborn and child health, much potential exists for scaling up nutrition promotion and therapeutic interventions using such platforms, hence integrating the two at point-of-service delivery. Community health workers have the potential to improve community trust and engagement, as well as enhance the overall health workforce (Perry *et al.*, 2016). They also can play an important role in the management of serious childhood illnesses, including malnutrition (Perry *et al.*, 2014). However, implementation of such programmes involves unique combinations and sequencing of health system policies, actions and advocacy, requiring both political will and adequate resources—two factors identified to be ‘most important’ lessons learned in this work. Such community-based programmes also need evidence-informed planning, a rights-based framework for engagement of communities and other sectoral involvement (e.g. Gender) (UNICEF, 2013). Those requisites are not insurmountable: this model has been successful to improve health services utilization in Ethiopia, Kenya and Senegal, e.g. (Kung'u *et al.*, 2017).

### Strengths and limitations

This study had several important strengths. First, it included methodological triangulation, using both in-depth interviews, pile sort data and participatory workshops to draw conclusions (Hussein, 2015). Second, it had an iterative and emergent design, a strength of qualitative research in general and a design aspect of this study that enabled workshop findings to build off those of interviews previously collected (Marshall and Rossman, 2014). Third, it included the perspectives of diverse stakeholders, including individuals from government, civil society, organizations and the community. Building consensus around key questions considering the perspectives of participants from different stakeholder groups gives us more confidence in our final interpretations and lends itself better to policy planning.

Some areas of this study could be improved. First, this study would benefit from a follow-up phase of in-depth interviews among purposefully sampled participants from the workshops, e.g. among those who held dissenting viewpoints or key informants to help clarify and interpret results. Such a phase would also allow us to elucidate the specific characteristics of each challenge, factor and lessons learned (Table 3) for more targeted follow up strategies tailored to each country context. Also, the sample size for the MDS analysis of pile sort data was smaller than desired; larger samples may have resulted in more stable results with less stress among items (Weller and Romney, 1988).

## Conclusion

Infectious disease outbreaks pose major challenges to population health and nutrition. The key challenges and lessons learned identified in this study, while discussed in light of nutrition, reflect larger systemic weaknesses indicative of two fragile health systems. No health system can be fully prepared for every threat to global health security. However, these study results, which contribute to a growing literature base of key lessons learned (Heymann *et al.*, 2015), may help inform health system strengthening and better emergency preparedness. Guinea and Sierra Leone offer rare insights into the challenges and opportunities that exist for the humanitarian nutrition sector in the context of infectious disease outbreaks.
